# A Review of Ophthalmic Complications in Inflammatory Bowel Diseases

**DOI:** 10.3390/jcm11247457

**Published:** 2022-12-15

**Authors:** Wiktoria Pytrus, Katarzyna Akutko, Tomasz Pytrus, Anna Turno-Kręcicka

**Affiliations:** 1 Ophthalmonology Clinical Centre SPEKTRUM, 53-334 Wroclaw, Poland; 2 2nd Department and Clinic of Paediatrics, Gastroenterology and Nutrition, Wroclaw Medical University, 50-369 Wroclaw, Poland; 3 Clinical Department of Ophthalmology, Wroclaw Medical University, 50-556 Wroclaw, Poland

**Keywords:** inflammatory bowel disease, extraintestinal manifestation, ocular involvement

## Abstract

Inflammatory bowel diseases (IBD), including Crohn’s disease (CD) and ulcerative colitis (UC), are chronic immune-mediated conditions caused by various polygenic and environmental factors. Clinical manifestations of IBD primarily occur in the gastrointestinal tract, but many patients are affected by extraintestinal complications, including eye diseases. Ocular disorders are the third most common extraintestinal manifestation (EIM), following musculoskeletal and mucocutaneous involvement. Episcleritis, frequently occurring in IBD patients, may be associated with exacerbation of the intestinal disease. Uveitis does not correlate with IBD activity but may be related to the presence of other EIMs, particularly erythema nodosum and peripheral arthritis. Early detection and specific therapy of ocular manifestations of IBD are fundamental to avoiding sight-threatening complications. Therefore, ophthalmic evaluation should be performed in all IBD patients. Systemic corticosteroids or immunosuppressants may be inevitable in severe cases to control ocular inflammation. Persistent and relapsing conditions usually respond well to TNF-α-inhibitors. Interdisciplinary cooperation between gastroenterologists and ophthalmologists is fundamental in initiating the appropriate treatment for patients.

## 1. Introduction

Inflammatory bowel diseases (IBD) are chronic diseases of the gastrointestinal tract of multifactorial etiology. IBD include Crohn′s disease (CD), ulcerative colitis (UC), and indeterminate colitis (IC). For several decades, an increasing incidence of IBD in both adults and children has been observed, especially in the Western world but also in developing countries [1,2,3]. The etiology of IBD is complex and not fully understood. The intestinal inflammation develops due to an interaction of genetic, local, immunological, and environmental factors (intestinal microflora contribution). More than 150 different genetic mutations responsible for the development of IBD have been reported [4,5,6,7,8]. The most crucial genes regulate the natural immune response, autophagy, production of inflammatory cytokines (NOD2 /CARD15, AT G16 L1, IL23R), or function of the intestinal epithelium (HNF4A, GNA12) [9,10]. Uncommon monogenous changes can be responsible for an earlier development of IBD (<6.y.-early onset IBD) and of a more severe and aggressive course [11]. So far, it has not been possible to assess the cause of the development of early IBD symptoms. However, association research of the entire genome (GWAS) and monogenic IBD research allowed the identification of over 50 genetic mutations predisposing carriers to the development of early IBD [12]. The overall global incidence of IBD, for both UC and CD, is estimated at 5–15/100,000 person years [13]. The highest incidence of both UC and CD is found in North America and Europe. Recently, a rising occurrence rate of IBD has been noted in Eastern Europe. IBD are becoming more common in newly industrialized countries in Asia, South America, and the Middle East [14,15]. The region of the world with the highest estimated incidence of UC (57.9/100,000) is Northern Europe. The highest estimated CD incidence (29.3/100,000) was recorded in Canada [16]. Epidemiological data suggest that in the coming years there will be no further increase in incidence in the Western world [13,17]. In contrast with Europe and North America, the incidence of IBD in African, Asian, and South American countries has been low in the last century. However, current epidemiological data show that in recent decades, an increase in the incidence of IBD has been observed in developing countries, both in South America (in 1988–2012, the incidence in Brazil increased by 11.1%) and Asia (in 1998–2008, the incidence in Taiwan increased by 4.0%). In both of those regions of the world, the peak incidence of IBD has not yet been reached [13].

The most common symptoms of IBD include diarrhea, abdominal pain, weight loss, and fatigue [18]. Diarrhea often contains an admixture of pathological blood or mucus and occurs at night. However, it should be emphasized that constipation may occur in some patients, especially those with rectal UC. Often, especially in UC, abdominal pain is characterized by increased pressure on the stools or pain in the left lower abdominal quadrant. Pain in the lower-right quadrant is common in the course of CD. Additionally, nausea and vomiting are more common in CD [19,20].

Though clinical manifestations of IBD are mainly located in the gastrointestinal tract, about 25% of patients present with different disorders which are unrelated to the gastrointestinal system. These symptoms are defined as extraintestinal manifestations (EIMs) of IBD and may involve any organ. The most common EIMs involve musculoskeletal disorders, mucocutaneous as well as ocular pathology, in that order. The prevalence of all EIMs is diverse and varies between 12–35% in UC and 20–25% in CD [18,21,22]. The genesis of EIMs is not fully understood, though it is probably associated with the immune reaction against antigens that enter the bloodstream through the damaged intestinal barrier, causing the deposition of antigen–antibody complexes in different tissues outside the gastrointestinal tract [23,24]. The incidence of extraintestinal manifestations in UC as opposed to CD is debatable. The majority of studies report a higher rate of EIMs in patients with CD. A few studies, however, show that the extraintestinal symptoms are equally common in both types of IBD. Some EIMs are associated with IBD flare (arthritis, erythema nodosum, pyoderma gangrenosum, and uveitis); others can develop regardless of the bowel inflammation process (ankylosing spondylitis; primary biliary cirrhosis, or PBC; primary sclerosing cholangitis, or PSC; autoimmune hepatitis, or AIH). Apart from the most common locations of EIMs, there are numerous case reports describing sporadic involvement of the reproductive system or a rare polyneuropathy known as Melkersson–Rosenthal syndrome [22,23]. The effects of smoking on IBD have been studied in numerous studies. The exact mechanisms involved in the interaction of smoking and the course of IBD remain unclear. It has been shown that smoking is a negative factor in the development of CD, but it may have beneficial effects on the course of UC. There is a debate regarding smoking and extraintestinal manifestations (EIMs’) development in IBD. Smoking has been reported to be a risk factor for the development of EIMs in patients with UC and in patients with CD. In the study by Roberts et al., ocular EIMs were significantly more common among smokers compared to nonsmokers in UC, but not in CD patients [25].

Ocular complications in IBD patients can be primary, secondary, or accidental. Primary complications are directly related to IBD exacerbations and most often resolve after systemic treatment. The most common primary complications include scleritis, choroiditis, and keratopathy. Secondary complications include, for example, cataracts due to treatment with corticosteroids or dry eye syndrome due to hypovitaminosis A as a consequence of post-resection short bowel syndrome. Accidental complications are those that occur in the general population, independent of IBD, e.g., conjunctivitis [26]. King et al. have reported that patients with certain ophthalmic symptoms (anterior uveitis, episcleritis, or scleritis) are at a twofold greater risk of subsequently being diagnosed with IBD than matched patients without these symptoms. The risk was highest in those subjects who had been later diagnosed with CD. Clinicians should be aware of the potential risk of an IBD in patients with these ophthalmic conditions [27].

The main focus of the following paper is the review of ocular manifestations in IBD, including their characteristics, prevalence, and clinical significance.

## 2. Ocular Extraintestinal Manifestation

Ocular symptoms occur in 0.3–13% of all IBD cases; 1.6–5.4% in UC cases; and 3.5–6.8% in CD cases [21,27]. Eye disorders may be a presentation of the disease itself or drug related. The most frequent ocular manifestations reported in IBD patients include episcleritis (2–5%) and anterior uveitis (0.5–3.5%) [26,28]. Meta-analysis by Ottaviano et al. reported data on ocular extraintestinal manifestations prevalence in children. Overall prevalence of O-EIMs was 0.62–1.82%. Uveitis was the most common complication in children with IBD. Prevalence of O-EIMs in children is generally lower than in adults but may be underestimated because of the possibility of asymptomatic uveitis. Children with CD may be at increased risk of O-EIMs [29]. Episcleritis is thought to escalate in parallel with IBD flares and resolve with effective therapy for intestinal inflammation. In contrast, anterior uveitis progresses independently of IBD activity; it may occasionally be the first manifestation of bowel disease [30]. Female sex and coexisting arthritis or arthralgia are considered to be risk factors for developing ocular manifestations. Eye manifestations are strongly associated with HLA-B*27, B*58, and HLA-DRB1*0103 loci [27,30,31]. Taleban et al. also showed the association between the presence of ocular EIMs and the gene locus encoding RBM19 [32]. Smoking is well known to have a protective impact against the progression of UC. It has also been shown to prevent the relapse of ocular manifestations in UC patients [21].

## 3. Episcleritis

Episcleritis, the most common eye complication associated with IBD, is an index of the disease activity [26,33,34]. This self-limiting condition more commonly affects women. The inflammation process is limited to the radially oriented superficial episcleral vessels within Tenon’s capsule. Two forms are distinguished: simple episcleritis and nodular episcleritis. Clinical features include a sudden onset of discomfort, tearing with or without photophobia, and mild-to-moderate pain in the eye globe. Episcleritis takes a form of either sectoral (rarely diffuse) redness of the episclera or a red nodule arising from the episclera that blanches with a topical vasoconstrictor (10% phenylephrine eye drops) [33]. Visual acuity is not disturbed. There is no change in pupillary response to light and no corneal involvement [33,35].

In general, anti-inflammatory IBD treatment is usually sufficient to resolve episodes of episcleritis [35]. Topical treatment may be added, beginning with lubricants and cold compresses to soothe ocular tenderness and foreign body sensation [33,36]. The role of topical NSAIDs and corticosteroids is unclear [33,37]. In spite of the lack of evidence for the benefit of topical NSAIDs (such as diclofenac, ketorolac, nepafenac, and bromfenac), they are widely used. They are licensed for the management of perioperative conditions but not episcleritis. Topical corticosteroids may be useful in cases where prolonged treatment is needed [33]. Oral NSAIDs (diclofenac sodium, naproxen, and flurbiprofen) should be considered in severe or recurrent cases. However, they should be administered carefully and under the supervision of a gastroenterologist, as they can trigger or worsen intestinal inflammation. 

Episcleritis may be inaccurately diagnosed as conjunctivitis, a common condition which may occur in IBD patients simultaneously, but which is caused by different factors, such as bacterial/viral infection, allergy, or contact-lens overuse [33,35,37].

## 4. Scleritis

Scleritis, a potentially blinding inflammation of the sclera, is much less common among IBD patients. It occurs in less than 1% of cases [33,38,39]. Unlike episcleritis, scleritis is not considered to be an index of IBD activity and may develop during a remission stage.

**Figure 1:**
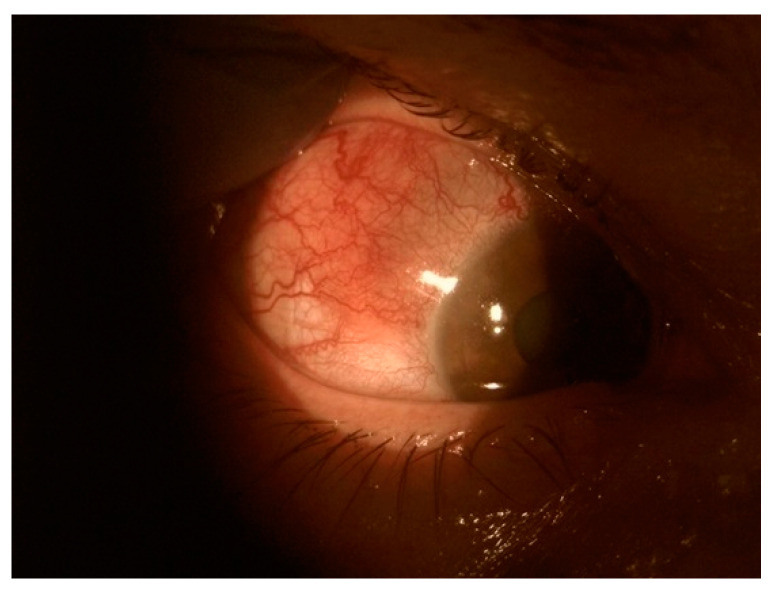
Scleritis.

Scleritis is most common in elderly patients. Hyperemia does not blanch with topical phenylephrine, since deep scleral vessels are involved (Figure 1). This condition is typically much more painful than episcleritis. The pain is usually constant, deep, and located behind the eye globe. It may radiate to the forehead, temporal region, and jaw. It typically worsens at night. Visual acuity is normal. There is no discharge or photophobia (unless the cornea is involved). There are many systemic diseases that predispose to the development of scleritis: rheumatoid arthritis, systemic vasculitis (including granulomatosis with polyangiitis), recurrent polychondritis, lupus erythematosus, systemic polyarteritis nodosa, Cogan’s syndrome, sarcoidosis, psoriatic arthritis, ankylosing spondylitis spondylitis, rosacea, atopy, gout, infections (e.g., syphilis; tuberculosis, varizella zoster virus) and trauma or surgery [26,28].

**Figure 2:**
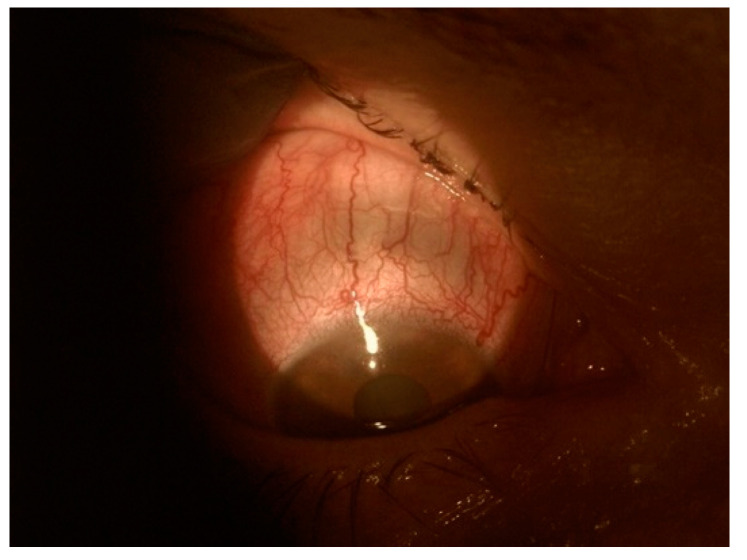
Translucent sclera with visible blue-black uveal tissue.

Necrotizing anterior scleritis occurs without inflammation (scleromalacia). Anterior scleritis can be non-necrotizing or necrotizing. Non-necrotizing anterior scleritis occurs in two forms: diffuse and nodular. Corneal involvement is more common in patients with nodular and necrotizing scleritis. It manifests as stromal keratitis and is frequently located near the limbus. Corneal neovascularization may develop in later stages. Necrotizing anterior scleritis with inflammation is characterized by white avascular areas surrounded by the injected sclera. If scleral necrosis develops, the tissue becomes translucent, showing the blue-black uveal tissue (Figure 2).

**Figure 3:**
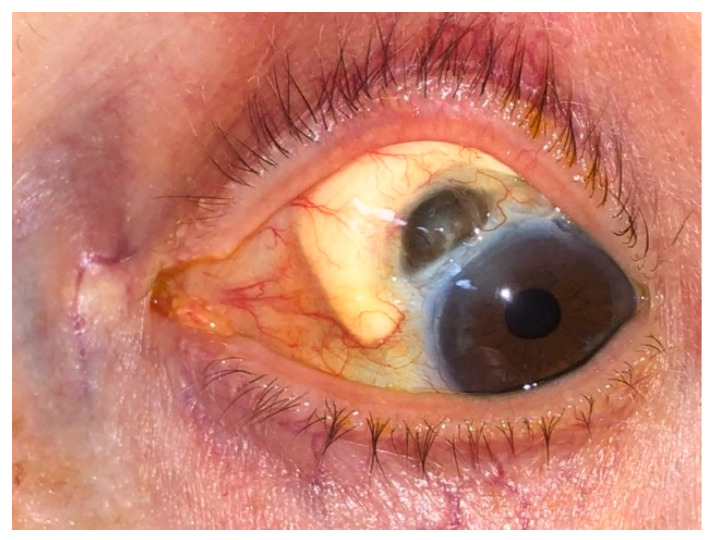
Scleromalacia perforans (treated with scleral allo-graft).

Necrotizing anterior scleritis without inflammation (scleromalacia perforans) is typically associated with severe chronic seropositive rheumatic arthritis (Figure 3) [33]. 

Posterior scleritis, rarely associated with IBD, presents as mild-to-severe deep retrobulbar pain (radiating to the brow or jaw). The ocular globe usually remains calm and white. However, lid edema, proptosis, and restricted mobility can be present. Visual acuity can be decreased due to retinal and choroidal involvement (choroidal folds, annular choroidal detachment, exudative retinal detachment, macular edema, or optic disc edema). A hypermetropic shift due to retinal edema is possible. Scleral inflammation involves the posterior segment and can be seen in the B-scan ultrasound as a T-sign (scleral thickening with fluid in the Tenon’s space) [28,33]. Systemic steroids or immunosuppression should be rapidly administered. Steroids are considered to be the rescue therapy (e.g., prednisolone 1 mg/kg/d, tapering down). Maintenance therapy requires immunosuppressants to prevent recurrence and treat the underlying bowel disease.

This inflammation involves the uveal tract itself (iris, ciliary body, and choroid) and the surrounding structures, namely the retina, vitreous, and optic nerve). It is usually divided into anterior uveitis (iritis and iridocyclitis), intermediate uveitis (vitreous involvement), and posterior uveitis (retinal involvement). When the inflammation involves all the uveal parts, it is called panuveitis [33,40].

One of the most common eye complications in IBD patients is uveitis. This condition can cause poor vision and even blindness [41]. The typical presentation of uveitis in IBD patients is an acute anterior uveitis, a non-granulomatous type of uveitis (seen in up to 5% of IBD patients) which is usually bilateral and persistent. The strongest independent risk factor for the development of uveitis in patients with CD or UC is the coexistence of other EIMs, mainly arthritis and erythema nodosum. In addition, the use of immunosuppressants in patients with inflammatory bowel disease is sometimes required to treat uveitis. This supports the idea that uveitis might be considered a marker of a severe disease course [42].

HLA-B27 positive patients are more likely to develop acute iritis, CD, and sacroiliitis [24,43]. Biedermann et al. showed that uveitis in CD patients has been linked with a positive family history of IBD [42].

Clinical features of anterior uveitis include pain, photophobia, redness, and blurred vision. Slit lamp examination reveals circumlimbal hyperemia, keratic precipitates, anterior chamber flare and cells, posterior synechiae, and anterior vitreous cells. Treatment of all cases of anterior uveitis requires topical corticosteroids (e.g., 0.1% dexamethasone, initially every 30–60 min, depending on response) in combination with topical cycloplegics (e.g., tropicamide 1% three times daily, cyclopentolate 1% three times daily or atropine 1% three times daily) to prevent contraction of the ciliary body causing eye pain and the formation of posterior synechiae. In more severe or recurrent cases, periocular injections or the systemic use of corticosteroids must be taken into consideration. The choice of immunosuppressive therapy must be discussed with a gastroenterologist, especially if the patient has another EIM.

## 5. Keratopathy

Corneal disorders in IBD were described in 1925 by Crohn, who reported two patients with keratomalacia and xerophthalmia due to presumed vitamin A deficiency [44].

Scleritis-related keratopathy usually occurs in the same area as or directly next to active scleritis. This condition can occur in several clinical forms which can vary in severity from mild to severe. Their proper differentiation is important because they require different procedures and have a different prognosis. It is important to make an appropriate diagnosis and start treatment to prevent complications such as significant deterioration of vision or perforation. Clinical types of peripheral keratopathy associated with scleritis include peripheral ulcerative keratitis (PUK), stromal keratitis, and peripheral corneal thinning [45].

**Figure 4:**
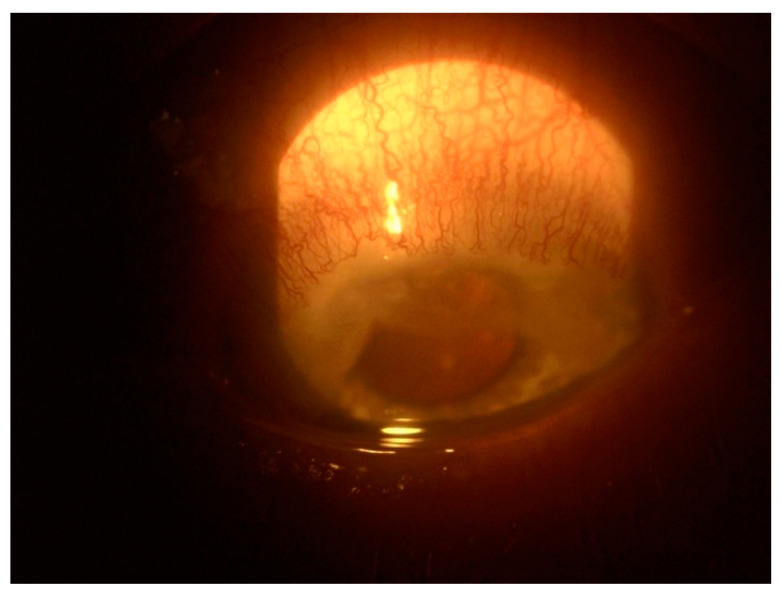
Peripheral ulcerative keratitis.

PUK is a sight-threatening form of keratitis which is frequently associated with underlying systemic disease. Clinical features include uni-/bilateral corneal ulceration with epithelial defect and stromal thinning in conjunction with inflammation at the limbus, as well as sectoral or diffuse scleritis (Figure 4). Infliximab has been shown to produce a rapid suppression of corneal inflammation, pain, and keratolysis in PUK associated with CD [46].

Other forms of corneal disorders are infiltrates and scar formations, which may occur in all corneal layers: epithelium, stroma, and endothelium. They do not decrease visual acuity when located outside the visual axis [47]. One study showed decreased corneal parameters such as lower corneal thickness in all IBD patients and reduced tear production in CD patients. Corneal thinning and dry eye disease (DED) may also be magnified by immunosuppressive therapy and anti-inflammatory drugs, such as 5-aminosalicylic acid [44]. Another study showed a higher incidence rate of DED among CD compared to UC patients and healthy controls [48]. Salzmann nodules, a slowly progressive corneal degeneration resulting from the replacement of the Bowman’s layer by the eosinophilic material, were reported as a distinct clinical sign of ocular involvement in CD [49,50]. IBD are a relative contraindicator to perform laser refractive surgery. Necrotizing keratitis was reported in patients with inactive CD who had been treated with LASIK and PRK procedures. Stromal inflammation developed during follow-up and had to be intensively treated with systemic and topical steroids [51].

## 6. Rare Ocular Findings in Patients with IBD

Although ocular involvement in IBD patients usually concerns the anterior part of the eye, there are many case reports and case series of other ocular manifestations [21]. These cover orbital inflammatory syndrome (dacryoadenitis, palpebral ptosis, lid margin ulcers, orbital myositis, and ocular myasthenia graves), retinal vasculitis (central serous chorioretinopathy, serpiginous chorioretinopathy, acute macular neuroretinopathy, and macular edema), retinal vascular occlusions, retinal neovascularization, optic neuritis, and pseudotumor cerebri [26,28].

## 7. Ocular Disorders Related to IBD Treatment

Posterior subcapsular cataracts and glaucoma in steroid responders are well known side effects of the prolonged use of corticosteroids and severe ocular inflammation [31,52]. Corneal immune infiltrates and diffuse retinopathy have been described as related to adalimumab treatment [52]. Infliximab can cause anterior optic neuropathy and retinal vein thrombosis. The systemic use of methotrexate and its presence in tears may result in irritation of the conjunctiva and cornea. Cyclosporine has been reported as a possible cause of rare optic neuropathy, ophthalmoplegia and nystagmus [26]. Surprisingly, anti-TNF-α-inhibitors (etanercept, infliximab, and adalimumab), which are supposed to eliminate inflammation, are occasionally considered a trigger for uveitis. They can also be an uncommon cause of drug-induced optic neuropathy and ophthalmoplegia [53].

## 8. Treatment

Episcleritis as a benign condition rarely requires any specific treatment, but it may be a sensitive indicator of IBD flares, as the two conditions tend to appear simultaneously. In the majority of IBD patients, episcleritis resolves with effective control of intestinal inflammation [26,28,38]. TNF-α-inhibitors (mainly infliximab) have been demonstrated to be effective in relapsing cases associated with IBD [38,39].

In contrast, uveitis may occur independently of intestinal disease exacerbation and may have a devious and persistent course. Thus, it demands prompt and intensive therapy to avoid potential complications such as glaucoma, band keratopathy, persistent hypotony, refractory macular edema, chronic ocular pain, and phthisis bulbi [21]. Topical steroids and topical cycloplegics are the initial treatment, but according to the degree of inflammation in the eye, periocular steroid injections or systemic steroids may be unavoidable. Uveitis with a recurrent course or prolonged duration requires immunosuppressive therapy to avoid the negative influence of extended corticosteroid use. The decision about the most suitable immunosuppressive agent requires an interdisciplinary approach, particularly when a patient develops another extraintestinal manifestation of IBD. Table 1 summarizes the most common ocular EIMs and their treatment options.

A number of immunosuppressants, such as cyclosporine, thiopurines, and methotrexate, have been proved to be effective in the management of both IBD and ocular EIMs [38,39]. TNF-α-inhibitors (infliximab and adalimumab) are reserved for refractory ocular inflammation cases or in the presence of other EIMs. Adalimumab has been approved for the treatment of IBD and is considered efficacious in severe cases of uveitis [26].

## 9. Conclusions

Ocular involvement in IBD is a rare extraintestinal manifestation, but it may be critical because of its potential sight-threatening complications if not treated promptly and accurately. In addition, episcleritis in IBD is an important indicator of bowel disease activity. Uveitis may precede the symptoms from the gastrointestinal tract, so it is crucial to ask the patient about other problems (fever, abdominal pain, weight loss, or diarrhea). Ophthalmic examination should be recommended to all IBD patients, since asymptomatic inflammation of ocular tissues is possible. Patients should be informed of the ocular side effects of prolonged corticosteroid use such as cataracts and glaucoma. Moreover, some anti-inflammatory medications used in IBD therapy may cause ophthalmic adverse effects. A collaborative approach is essential in the management of these patients.

## Figures and Tables

**Table 1:** Ophthalmic manifestations in inflammatory bowel diseases.

Episcleritis	Scleritis	Uveitis
Indicates IBD flares.	May develop during a remission stage.	May develop during a remission stage.
Self-limiting condition.	Potentially blinding inflammation	May have devious and persistent course.
Resolves with IBD treatment.	Systemic steroids or immunosuppression.	Topical steroids and topical cycloplegics (recurrent cases require systemic steroids or immunosuppression).
Good prognosis.	Uncertain prognosis.	Uncertain prognosis.
